# SSVEP Extraction Based on the Similarity of Background EEG

**DOI:** 10.1371/journal.pone.0093884

**Published:** 2014-04-07

**Authors:** Zhenghua Wu

**Affiliations:** 1 School of Computer Science and Engineering, University of Electronic Science and Technology of China, Chengdu, Sichuan, China; 2 Key Laboratory for Neuro Information of Ministry of Education, School of Life Science and Technology, University of Electronic Science and Technology of China, Chengdu, Sichuan, China; University of Rome, Italy

## Abstract

Steady-state Visual Evoked Potential (SSVEP) outperforms the other types of ERPs for Brain-computer Interface (BCI), and thus it is widely employed. In order to apply SSVEP-based BCI to real life situations, it is important to improve the accuracy and transfer rate of the system. Aimed at this target, many SSVEP extraction methods have been proposed. All these methods are based directly on the properties of SSVEP, such as power and phase. In this study, we first filtered out the target frequencies from the original EEG to get a new signal and then computed the similarity between the original EEG and the new signal. Based on this similarity, SSVEP in the original EEG can be identified. This method is referred to as SOB (Similarity of Background). The SOB method is used to detect SSVEP in 1s-length and 3s-length EEG segments respectively. The accuracy of detection is compared with its peers computed by the widely-used Power Spectrum (PS) method and the Canonical Coefficient (CC) method. The comparison results illustrate that the SOB method can lead to a higher accuracy than the PS method and CC method when detecting a short period SSVEP signal.

## Introduction

A Brain-computer Interface (BCI) is an alternative communication channel used to connect the brain to external electronic devices. This technique is important in assisting, augmenting or repairing human cognitive or sensory-motor functions [1], [2]. Recently, non-invasive BCI employing Steady-state Visual Evoked Potential (SSVEP), as a measurement, has gained increasing attention due to SSVEP’s strong immunity to nuisance noises, such as eye and body movement [3], [4], [5], [6], [7]. In some typical SSVEP-based BCI communication paradigms, visual stimuli may flicker at different frequencies on the monitor in front of a subject [5], [7], [8], [9]. The subject can select a particular stimulus by visually staring at it. Multiple frequencies can be used simultaneously to characterize a complicated system. Many studies have shown that SSVEP-based BCI has advantages in implementation [1], [2], [3], [10], [11], such as high accuracy, noise immunity, short detection time and a relatively high Information-transfer Rate (ITR). The development of SSVEP-based BCI opens up the prospect of allowing disabled patients to effectively improve control over external devices such as monitors, prostheses and wheelchairs [2], [6], [12], [13], [14], [15].

The performance of the BCI can be characterized using ITR, which is determined by three factors: accuracy, speed and the target amount [5], [16]. To increase the ITR of SSVEP-based BCI for practical scenarios, many SSVEP extraction methods have been proposed. The most widely used method is the so-called Power Spectrum Method (PS) [3], [5], [6]. In the PS method, Fast Fourier Transform (FFT) is employed to estimate the power spectrum of the EEG from which the power of a particular frequency component can be computed. The power of a certain frequency in spontaneous EEG is used as a threshold for the corresponding SSVEP detection in evoked EEG. The power of a specific frequency in evoked EEG surpassing the threshold, indicates SSVEP. The detection normally lasts a few seconds in PS-based analysis. Online Autoregressive (AR) Model parameters estimation was also used for SSVEP extraction [17], [18]. The precision of this AR based method requires the signals to be stationary over a time period of 4–5 s. As such, this AR method is not suitable in a real-time system. Independent Component Analysis (ICA) has also been used to isolate SSVEP from evoked EEG [19]. Based on wavelet analysis, we proposed a method called the Steady Coefficient (SC) Method in 2008 [20]. In this method, the stability of a specific frequency is used as an indicator of SSVEP. If the steady coefficient within a period holds at the same level as that in spontaneous EEG, then there is no indication of SSVEP in evoked EEG. Otherwise, SSVEP is included in evoked EEG.

Few works employ ICA or other Time-frequency Analysis Methods for SSVEP extraction, because the temporal resolution of these methods is not high enough for a real-time system. In 2007, the computation of a correlation coefficient between a series of stimulus harmonics and the EEG was conducted [21], with this method being referred to as the Canonical Coefficient (CC) Method. Because of its high temporal resolution, the CC method has been used in a number of real-time cases in the past. However, there is a limitation to the CC method. Because the correlation coefficient is very sensitive to the initial phase of SSVEP, normally many channels are used simultaneously in the CC method in order to avoid this drawback, and this increases the complexity of the system. All of the methods listed above are related to the power of SSVEP. In other methods, phase is used to detect SSVEP [10, 22, 23, 24]. The methods above are all directly related to SSVEP power and phase and can therefore be considered as direct methods.

The detection of specific frequencies can also be ascertained by using an indirect method. For example, an original signal which is composed of varying frequencies can be transformed into a new signal by filtering out the target frequency. By comparing similarities between the original signal and the new signal, it can be concluded indirectly that the target frequency is, or is not, strong enough. Obviously, EEG is a kind of signal composed of many frequency components. When a steady-repetitive stimulus emerges in the visual field, the corresponding frequencies (or defined as target frequencies) in the EEG are enhanced. In consequence, it is possible to compare the similarities between the original EEG and the remaining which filtered out target frequencies. If the correlation coefficient is above a certain threshold, suggesting that there is no clear influence on the original EEG through filtering, it can be concluded that the level of power corresponding to the target frequencies is low. In other words, there is no SSVEP included in the EEG. Alternatively, if the correlation coefficient is below a certain threshold, it can be concluded that there is a valid SSVEP in the original EEG. This method is termed the Similarity of Background (SOB) Method. Although the SOB method and the CC method both make use of the computing correlation coefficient, the SOB is an indirect method and is insensitive to the initial phase of SSVEP, while the CC method is a direct method, sensitive to the initial phase of SSVEP. In order to evaluate the validity of the SOB method, the SOB method, the PS method and the CC method are all used to detect SSVEP in 1s-length and 3s-length EEG segments, respectively. When detection accuracy is compared between methods, the SOB method has been shown to have a greater accuracy than the widely used PS and CC methods for short periods, such as 1s-length under the situation of middle or weak SSVEP. This comparative result suggests that the SOB method is a good candidate for real-time SSVEP-based BCI applications.

## Methods

### 2.1 Statement of Ethics

This study was approved by the Human Research and Ethics Committee of the University of Electronic Science and Technology of China. Before the experiment, all the subjects were told the purpose and procedure of the experiment in detail and signed a consent form. These forms were approved by the University of Electronic Science and Technology of China Ethics Committee Data Acquisition.

### 2.2 Stimulus Design

Eleven subjects participated in this experiment. They had normal or corrected normal visual acuity**.** A high luminance LED was used as the SSVEP stimulator and the subjects were seated 60 cm in front of the LED. The LED was activated by a series of square waves with a 1∶1 duty cycle of 33.33, 25, 16.67, 12.5, 8.33, and 6.25 Hz, respectively. During the experiment, the subjects were asked to blink normally and to avoid moving their bodies. EEG was recorded using a 129-channel EEG system referenced at Cz (Electrical Geodesics Incorporated (EGI) amplifier system 200, USA). The sample rate was set to 250 Hz. Electrode impedance was kept below 10 kΩ, and salt water was dropped into the electrode periodically in order to retain quality contact with the subject’s scalp. A 60s-length spontaneous EEG was recorded first and used to build a threshold. A 60s-length evoked EEG was then recorded for each stimulus. The data was stored on a hard disk for offline processing. Since there is only one channel used for SSVEP extraction in the SOB or PS methods, a signal electrode with strong SSVEP is very important. According to the suggestions of some previous electrode selection studies [25], [26], Oz (No.76 electrode) was selected as the signal electrode for all stimuli. In order to compare the SOB method with the CC method under the same situation, only one signal channel (No. 76 electrode) was used in the CC method for this work.

### 2.3 Methodology

SSVEP power concentrates at the stimulus frequency and its harmonics, and stimulus frequency adopted in the SSVEP experiment is usual higher than 5 Hz, while the frequency of the noise caused by eye or body movement is far lower than 5 Hz, so SSVEP has relative immunity to the noise such as eye or body movement [5], [7], no pre-processing method, such as eliminating eye movement was adopted in this work. The frequency spectrum of 60s-length evoked EEG at the signal electrode was checked first for each stimulus. If a peak clearly identified at the frequency ‘f’ is higher than 3 times of the level of power averaging over the range from ‘f–2’ Hz to ‘f+2’ Hz, it suggests that the evoked EEGs are valid and can be decoded by the different methods, which will be identified below.

The SOB method can be described in three steps as follows. For a fixed length, EEG segment ‘S’, is first processed by FFT to obtain a spectrum. For high frequency stimuli such as 33.33 Hz and 25 Hz, frequencies below 11 Hz in the spectrum are filted out to cancel background EEG. For the other frequency stimuli such as 16.67, 12.5, 8.33 and 6.25 Hz, the components below 5 Hz in the spectrum are filtered out to avoid influence by the strong low frequency EEG and the components between 9 Hz and 11 Hz in the spectrum are filtered out to cancel the strong background α signal. This pre-processed spectrum is then processed using Inverse Fast Fourier Transform (IFFT) to get a new ‘S1’ signal in the time domain. The filter adopted in the SOB method is based on the frequency domain and is simple to realize. Components can be filtered by setting the amplitude and phase of the components to zero in the frequency spectrum. After applying IFFT to the processed spectrum, the signal can be established in the time domain by filtering out specific components.

The pre-processed spectrum is further processed. For high frequency stimuli such as 33.33 Hz or 25 Hz, the pre-processed spectrum filters out only the components near the first harmonic, i.e. frequencies from ‘f1–0.5’ Hz to ‘f1+0.5’ Hz, where ‘f1’ stands for the first harmonic. For other frequency stimuli, the pre-processed spectrum filters out the components near the first and second harmonic, i.e. frequencies from ‘f1–0.5’ Hz to ‘f1+0.5’ Hz and from ‘f2–0.5’ Hz to ‘f2+0.5’ Hz, with ‘f1’ and ‘f2’ standing for the first and second harmonic respectively. This spectrum is then processed using IFFT to get a new ‘S2’ signal in the time domain.

**Figure 1:**
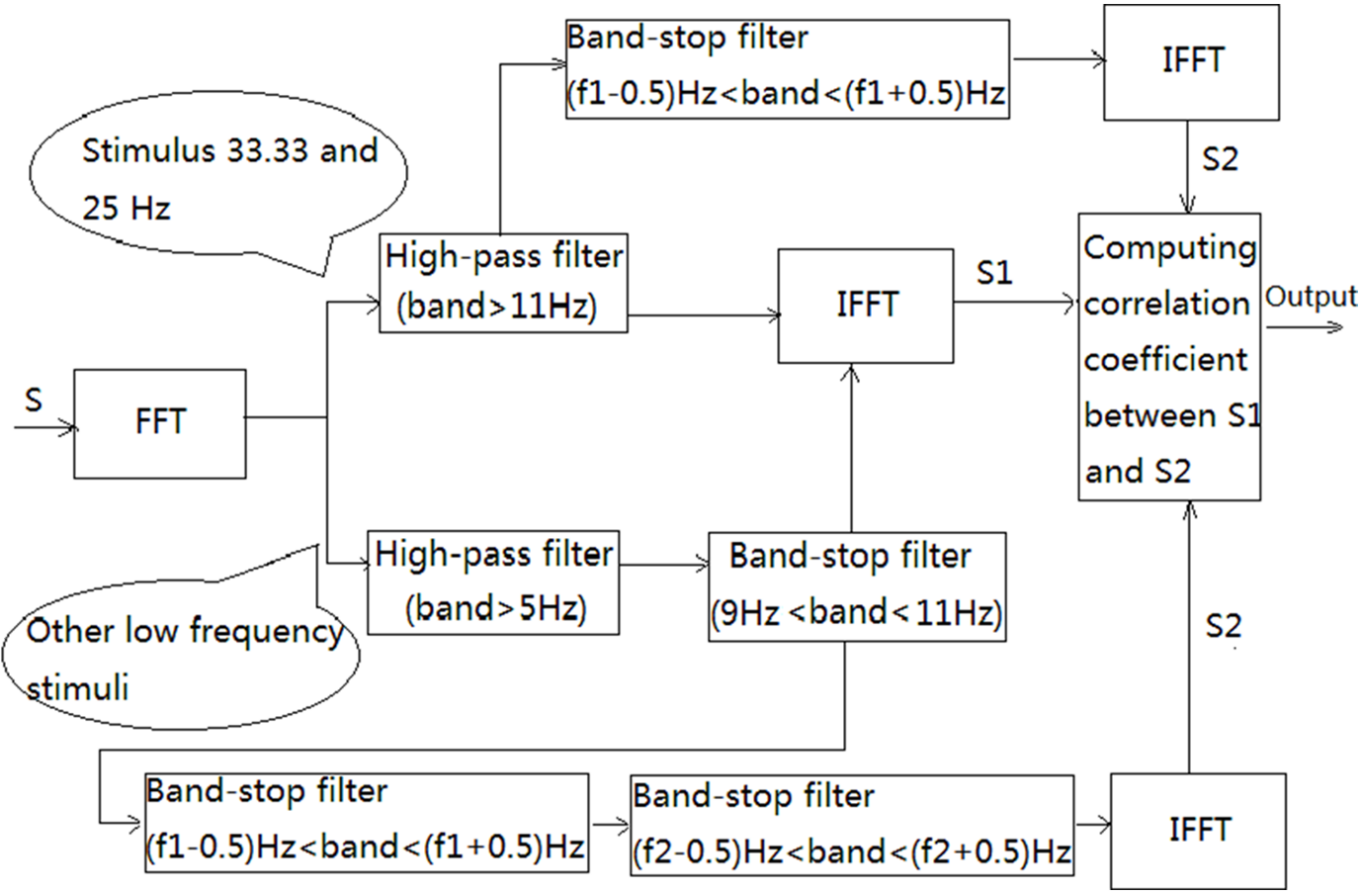
Detailed steps of the SOB method. ‘f1’ stands for the first harmonic of a stimulus and ‘f2’ stands for the second harmonic of a stimulus. The Band-stop filter, for example, ‘Band-stop filter (9 Hz<band<11 Hz)’, refers to the signal from the 9 Hz to 11 Hz filters.

The third step of SOB concerns calculating the correlation coefficient between the ‘S1’ and ‘S2’ signals, with the correlation coefficient being computed according to ‘corrcoef’ function in MATLAB. The transformation of FFT and IFFT are completed using the ‘fft’ and ‘ifft’ function in MATLAB. Figure 1 illustrates the detailed steps of the SOB method.

Before applying different methods to SSVEP detection, spontaneous EEG and different frequency evoked EEGs were divided into 60 segments of 1s-length. In the SOB and PS methods, FFT was adopted. In order to improve the frequency resolution, a ‘0’ series of 1s-length was added to the end of the EEG segments to get a 0.5 Hz frequency resolution. The technique of adding a ‘0’ series is widely applied in BCI studies [3], [5]. Two types of detection were utilized. The first shows whether SSVEP can be detected from a specific segment including a known frequency SSVEP. The second shows if other SSVEP frequencies can be detected from the same segment. This detection accuracy is called first type and second type accuracy. In the SSVEP frequency recognition, a correct detection means the first and second type of detection are both correct. Usually, the overall system detection accuracy is smaller than the first or second type accuracy. Specially, the best overall system detection accuracy can at best equal the worst of the two accuracy rates.

The SOB method was first applied on the 60 spontaneous EEG segments to get a series of correlation coefficients, which were used to build a threshold of SSVEP. The threshold for each frequency can be confirmed as follows: for a certain frequency, a hypothetical threshold was first used to test the corresponding coefficient in each spontaneous EEG segment and then adjusted continually until 90% of these coefficients (i.e. 54 coefficients) were bigger than the threshold. This means that the detection accuracy for spontaneous EEG is 90% at this frequency. Following this, six thresholds were built and the SOB method applied on every evoked EEG segment six times, one time for each frequency, resulting in six coefficients. Each coefficient is compared with its corresponding threshold to confirm the inclusion of a corresponding frequency SSVEP. If there is no coefficient smaller than the corresponding threshold, then the detection for this segment is wrong. If there are two or more coefficients smaller than the corresponding thresholds, then the detection of the segment is also wrong. The detection of this segment is correct, only if the coefficient of the known frequency in the evoked EEG segment is smaller than its corresponding threshold.

The PS method is then applied to these EEG segments. For a specific frequency ‘f’ Hz, the relative-power of ‘f’ Hz is computed as follows:


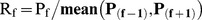
(1)

Where ‘R_f_’ stands for the relative-power of ‘f’ Hz, ‘P_f_’ stands for the absolute-power of ‘f’ Hz, ‘**mean**(P_(f-1)_, P_(f+1)_)’ stands for the average absolute-power from ‘f−1’ Hz to ‘f+1’ Hz.

For high frequency stimuli such as 33.33 Hz and 25 Hz, only the relative-power of the first harmonic is used as the indicator of SSVEP. For the other low frequency stimuli, the sum of relative-power of the first and second harmonic is used as the indicator of SSVEP. The threshold selection and SSVEP detection were similar to that of the SOB method. The PS method was first applied on the 60 spontaneous EEG segments to get a series of relative-power and then to get a series of indicators for different frequency SSVEP. The threshold for each frequency is confirmed as follows: for a certain frequency, a hypothetical threshold was first used to test the indicator of the corresponding frequency in each spontaneous EEG segment and then adjusted continually until 90% of these indicators (i.e. 54 indicators) were smaller than the threshold. This means that the detection accuracy for spontaneous EEG is 90% at this frequency. After this, six thresholds were built and the PS method applied to every evoked EEG segment six times, one time for each frequency, resulting in six indicators. Each indicator is compared to its corresponding threshold to confirm that a corresponding frequency SSVEP is included. If there is no indicator bigger than the corresponding threshold, the detection for this segment is wrong. If there are two or more indicators bigger than the corresponding thresholds, the detection for this segment is also inaccurate. Only if the indicator of the known frequency in the evoked EEG segment is bigger than its corresponding threshold is the detection correct.

**Figure 2:**
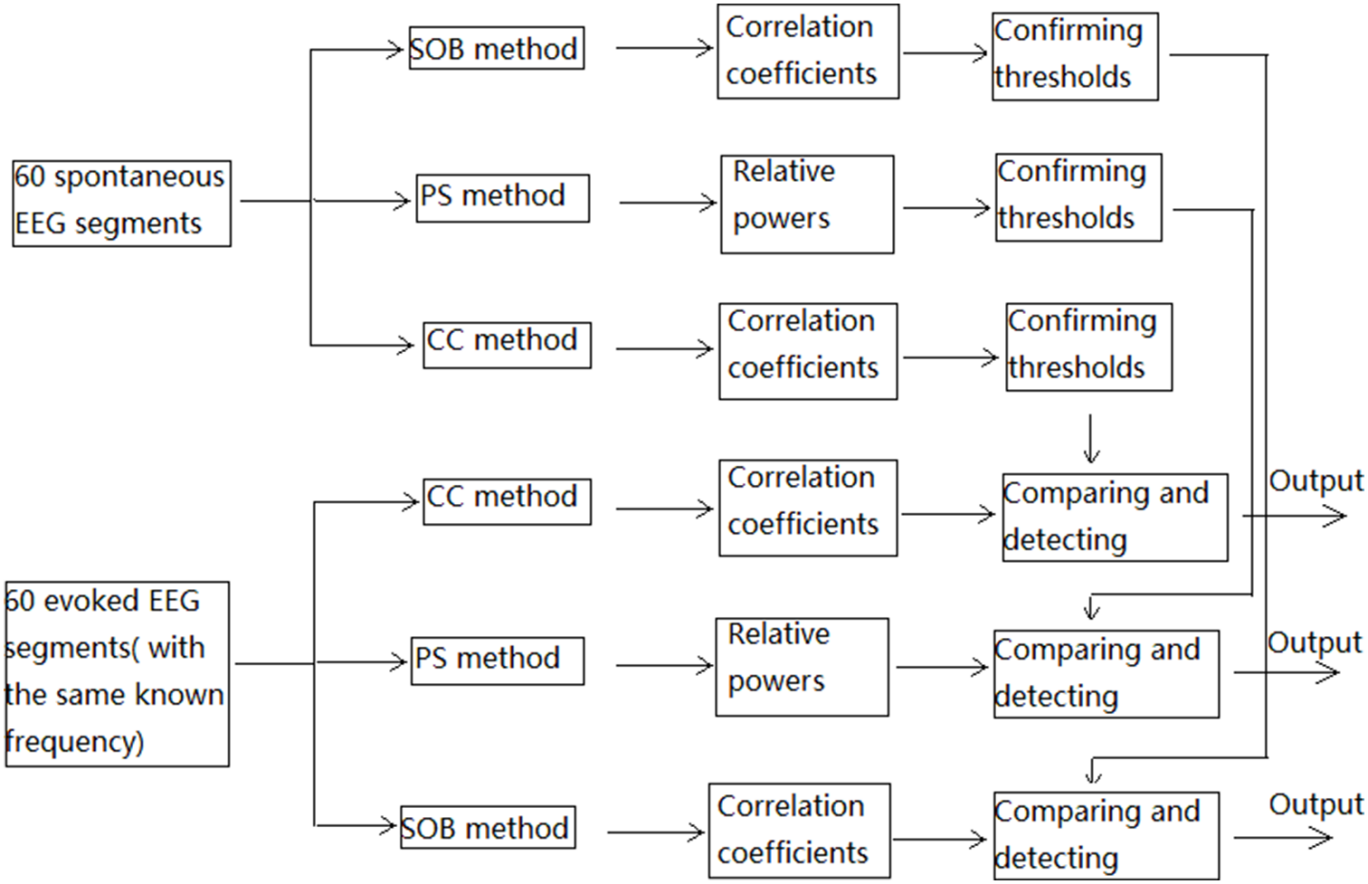
SSVEP detection steps for known frequency evoked EEG segments. ‘Output’ stands for the detection of an evoked EEG segment being accurate or inaccurate.

We applied the CC method to these EEG segments to detect SSVEP. For a certain frequency ‘f’ Hz stimulus, a series of sinusoidal signals of 1s-length with the same frequency ‘f’ Hz but a different initial phase were built first. The initial phase ‘φ’ is a series of estimated values, i.e. 0, (2*pi*1)/20, (2*pi*2)/20, …, (2*pi*19)/20 radian. The CC method was first applied to the spontaneous EEG segment. The correlation coefficient between each sinusoidal signal with different initial phase and the spontaneous EEG segment were computed, and the biggest coefficient selected as the correlation coefficient of the corresponding frequency for this segment. Thus, 6 correlation coefficients can be established for each segment. For high frequency stimuli such as 33.33 Hz and 25 Hz, only the coefficient of the first harmonic is used as an indicator of SSVEP. For the low frequency stimuli, the sum of coefficient of the first and second harmonic is used as the indicator of SSVEP. These indicators can be used to build the corresponding thresholds. For a specific frequency, a hypothetical threshold is first used to test the indicator in each spontaneous EEG segment. These were continually adjusted until only 90% of the indicators (i.e. 54 indicators) were smaller than the threshold, which meant that detection accuracy for spontaneous EEG was 90% at this frequency. After this, six thresholds were built and the CC method applied to every evoked EEG segment six times, one time for each frequency, resulting in six indicators. Each indicator is compared to its corresponding threshold to confirm whether a corresponding frequency SSVEP is included. If there is no indicator greater than the corresponding threshold, the detection for this segment is wrong. If there are two or more indicators bigger than the corresponding thresholds, the detection for this segment is wrong also. Only if the indicator of the known frequency in the evoked EEG segment is bigger than its corresponding threshold is the detection for this segment correct. Figure 2 illustrates the SSVEP detection steps for the three methods.

In order to understand the performance of different methods changing with the signal duration, the 60s-length spontaneous EEG and SSVEP were divided into 3s-length segments respectively. The similar steps as that for 1s-length segments were employed on these 3s-length segments. The detection accuracies of 3s-length segments were compared to those of 1s-length segments.

In order to evaluate the differences between the extraction methods, One-way Analysis of Variance (ANOVA) is used and the significance level ‘p’ is set to 0.05. A ‘p’ value smaller than 0.05 suggests that there is a significant difference between the two compared situations.

## Results

### 3.1 Comparing First Type Accuracy

After applying FFT to the 60s-length evoked EEG of each subject, a clear peak was found in the frequency spectrum for each stimulus, suggesting the successful evocation of SSVEP under each situation. Therefore, all evoked EEG can be used to detect SSVEP using different methods. This experiment illustrates a wide variety of SSVEP power across various subjects. For some subjects, all SSVEPs under every stimulus were strong and extracted easily with high accuracy using the PS, SOB and CC methods. For example, when the EEG segment lasted 1s, the average first-type accuracy of subject S1 was 92%, 95% and 85% for the PS, SOB and CC methods respectively, while its peer was 98%, 96% and 86% for the PS, SOB and CC methods respectively when the length of EEG segment was 3s. For some people, SSVEPs were strong for certain stimuli, while weak for others. This difference led to differentiated accuracy in detecting SSVEP. For example, subject S7 had a very different detection accuracy under different stimuli. For a 33.33 Hz stimulus, when the EEG segment lasted 1s, first type accuracy was 92% for PS, 99% for SOB and 87% for CC methods respectively, while its peer was 97% for PS, 98% for SOB and 88% for CC methods respectively when the length of EEG segment was 3s. While for a 25 Hz stimulus, when the EEG segment lasted 1s, first type of accuracy was 37% for PS, 47% for SOB and 44% for CC methods respectively, while its peer was 65% for PS, 50% for SOB and 46% for CC methods respectively when the length of EEG segment was 3s. For all eleven subjects, when the EEG segment was 1s-length, the overall averages of first type accuracy for PS, SOB and CC were 60%, 68% and 58%, respectively. When the EEG segment was 3s-length, the overall averages of first type accuracy for PS, SOB and CC were 82%, 72% and 60%, respectively. When the EEG segment was 1s-length, the ANOVA results between the PS and SOB methods were (F (1, 20) = 0.95, p = 0.34), which indicates that there is no significant difference between the two methods if all subjects are treated as one group. Likewise, the ANOVA result were (F (1, 20) = 1.9, p = 0.18) between the SOB and CC methods and (F (1, 20) = 1.47, p = 0.23) between the PS and CC methods, which indicates that there is no significant difference between the two methods if all subjects are treated as one group. When the EEG segment was with a length of 3s, the ANOVA results were (F (1, 20) = 1.3, p = 0.04) between the PS and SOB methods and (F (1, 20) = 15.1, p = 0) between the PS and CC methods, which means the accuracy of PS is significantly higher than that of SOB or CC; there was no significant difference of the detection accuracy between the SOB and CC (F (1, 20) = 2.9, p = 0.1).

**Figure 3:**
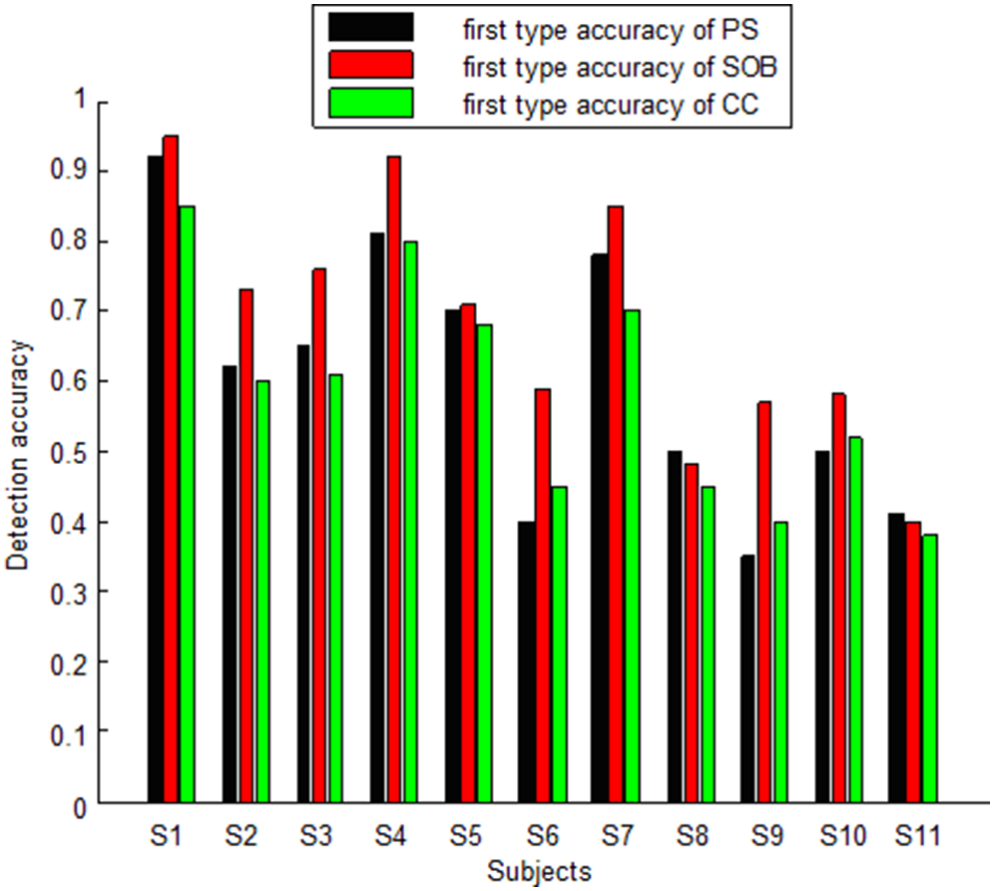
Average first type accuracy resulting from the PS, SOB and CC methods for every subject for 1s-length segments.

**Figure 4:**
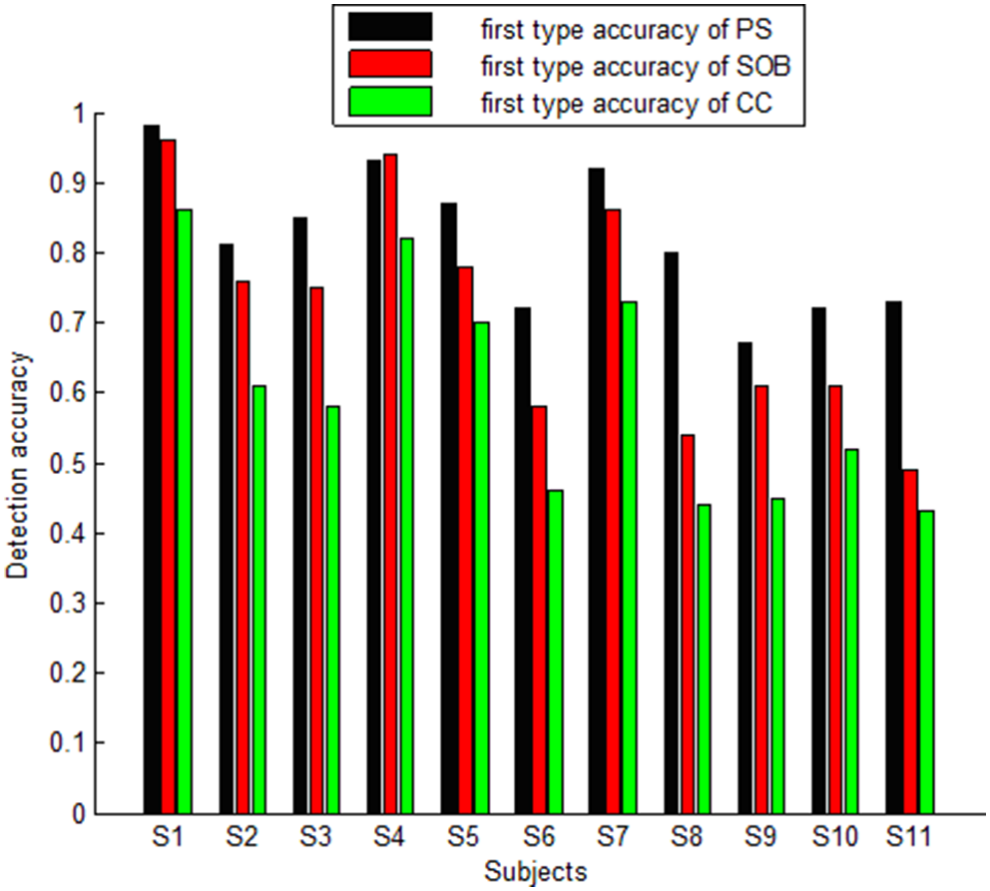
Average first type accuracy resulting from the PS, SOB and CC methods for every subject for 3s-length segments.

For 1s-length EEG segments, when dividing the subjects into three groups based on the detection accuracy, there was a big difference between the PS and SOB methods, and the SOB and CC methods. The ANOVA results for the group with accuracies higher than 85% were (F (1, 4) = 1.93, p = 0.24) between PS and SOB, and (F (1, 4) = 5.4, p = 0.08) between SOB and CC. This indicates that there is no significant difference between the PS and SOB methods or between the SOB and CC methods when SSVEP is prominent. However, the ANOVA results for the group with accuracies close to 70% were (F (1, 4) = 8.93, p = 0.04) between PS and SOB, and (F (1, 4) = 12.6, p = 0.02) between SOB and CC. This demonstrates a significant difference between the PS and SOB methods and the SOB and CC methods with reference to middle strength SSVEP. The ANOVA results for the group with accuracies around 55% is (F (1, 4) = 14.2, p = 0.02) between PS and SOB and (F (1, 4) = 12.2, p = 0.02) between SOB and CC. These results suggest that the differences between the methods are significant when SSVEP is weak. The ANOVA results between the PS and CC methods for the three groups of different accuracy were (F (1, 4) = 1.1, p = 0.29), (F (1, 4) = 1.3, p = 0.2) and (F (1, 4) = 1.6, p = 0.15) respectively. This illustrates that there is no significant difference between the PS and CC methods for first type detection under any situation. Fig. 3 shows the average first-type detection accuracy for every subject using the PS, SOB and CC methods for 1s-length segments. Fig. 4 shows the average first-type detection accuracy for each subject using the PS, SOB and CC methods for 3s-length segments.

Compared the first type detection accuracy of 1s-length segment to that of 3s-length segment, for the PS method, the first type detection accuracy of 3s-length segment is significantly higher than that of 1s-length segment (F (1, 20) = 10.1, p = 0.001); while for the SOB and CC method, the ANOVA results are (F (1, 20) = 0.01, p = 0.95) and (F (1, 20) = 0.01, p = 0.98) respectively, suggesting that there is no significant difference of the first type detection accuracy between the 1s-length and 3s-length segments for these two methods.

### 3.2 Comparing Second Type Accuracy

**Figure 5:**
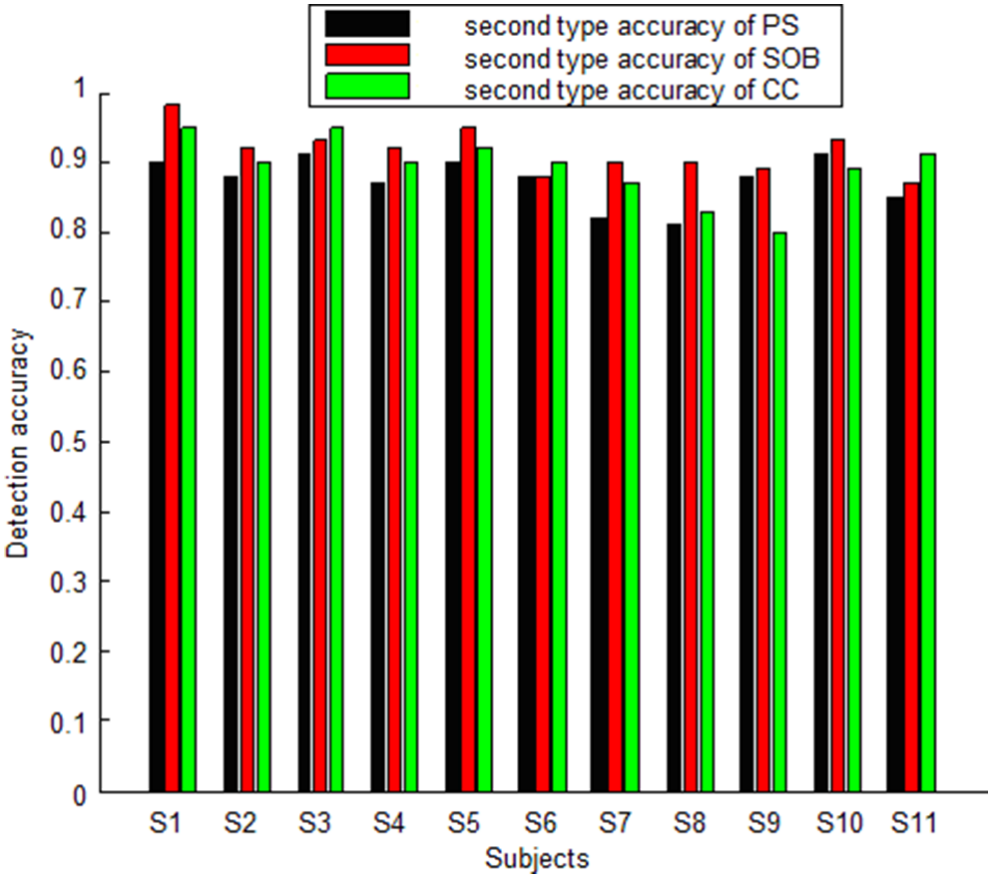
Average second type accuracy resulting from PS, SOB and CC methods for every subject for 1s-length segments.

**Figure 6:**
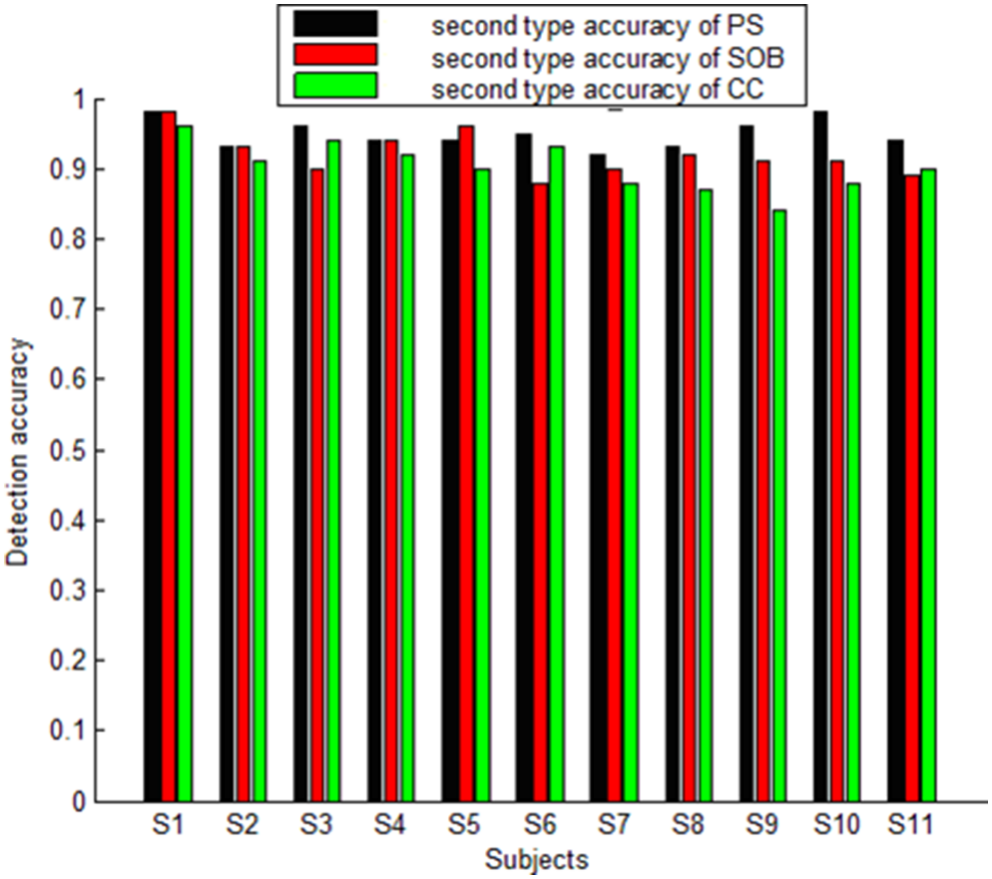
Average second type accuracy resulting from PS, SOB and CC methods for every subject for 3s-length segments.

Second type accuracy for the PS, SOB and CC methods are high for every subject under any stimulus. When the EEG segment lasts 1s, the average second type accuracy for the PS, SOB and CC methods is 87%, 92% and 89%, respectively. When the EEG segment is with length of 3s, the average second type accuracy for the PS, SOB and CC methods is 95%, 92% and 90%, respectively. When the EEG segment is 1s-length, the ANOVA results of (F (1, 20) = 8.3, p = 0.009) between the PS and SOB methods demonstrates that the second type accuracy of SOB is significantly higher than that of PS. However, the ANOVA results of (F (1, 20) = 1.83, p = 0.19) between the SOB and CC methods demonstrates that there is no significant difference between the two methods when calculating second type accuracy. Furthermore, the ANOVA results of (F (1, 20) = 2.8, p = 0.15) between the PS and CC methods demonstrates that there is no significant difference between the two methods when calculating second type accuracy. When the EEG segment is 3s-length, the ANOVA results of (F (1, 20) = 6.6, p = 0.02) between the PS and SOB methods and (F (1, 20) = 14.4, p = 0.001) between the PS and CC methods demonstrate that the second type accuracy of PS is significantly higher than that of the other two methods. However, the ANOVA result of (F (1, 20) = 1.56, p = 0.22) between the SOB and CC methods demonstrates that there is no significant difference between the two methods when calculating second type accuracy. Fig. 5 illustrates every subject’s average second type accuracy using the three methods for 1s-length segments. Fig. 6 illustrates every subject’s average second type accuracy using the three methods for 3s-length segments.

Compared the second type detection accuracy of 1s-length segment to that of 3s-length segment, for the PS method, the second type detection accuracy of 3s-length segment is significantly higher that of 1s-length segment (F (1, 20) = 4.7, p = 0.01); while for the SOB and CC method, the ANOVA results are (F (1, 20) = 0.33, p = 0.51) and (F (1, 20) = 0.41, p = 0.39) respectively, suggesting that there is no significant difference of the second type detection accuracy between the 1s-length and 3s-length segments for these two methods.

## Discussion

Three factors that likely have the largest influence on the detection accuracy of SSVEP are the SSVEP phase, SSVEP power and background EEG. The absolute or relative power of SSVEP is used as an indicator of SSVEP in the PS method. This indicator is irrelevant to the SSVEP phase. Therefore, the initial phase of SSVEP has no influence on the accuracy of the PS method.

Although the CC and SOB methods both make use of the correlation coefficient, there is a significant difference between them. Using the CC method directly to detect SSVEP only at one electrode, runs the risk of not choosing the highest correlation coefficient due to the fact that this method is very sensitive to the initial phase of SSVEP. In order to understand this more thoroughly, a sinusoidal signal can be added to a spontaneous EEG to obtain a simulative SSVEP signal. The correlation coefficient between the sinusoidal signal and the simulative SSVEP signal can then be calculated. If the starting point of the simulated SSVEP signal varies, (i.e. the initial phase of SSVEP varies), the correlation coefficient changes significantly. For example, when a 12 Hz sinusoidal signal of 1 μv is added to the spontaneous EEG at electrode No.76 for subject S1 to get a simulative SSVEP signal, by shifting the starting point of the simulated SSVEP signal, the correlation coefficient varies between the maximum 0.1372 and the minimum–0.1379. Using the CC method to extract 12 Hz at this channel results in many false detections, however, the power of 12 Hz in the simulative SSVEP signal holds almost at the same level. In this work, even though some estimated initial phases were used to compute the correlation coefficient, which can increase the coefficient to some extent, there were still limitations to obtaining the largest coefficient. In a real BCI system, where the subjects are randomly shifting their fixation point between flickers, the initial phase of each SSVEP frequency is unknown. If the correlation coefficient between the EEG at one electrode and the sinusoidal signal of the corresponding frequency is directly computed, detection accuracy will be low. Therefore, in studies in which the CC method was introduced to extract SSVEP [21], a few electrodes were used at the same time. Although this can improve the accuracy of the CC method, it increases the complexity of the BCI system, which is disadvantageous for a practical system.

The SOB approach has overcome the drawback of the phase sensitivity of the CC method. Under this approach, instead of directly computing the correlation coefficient between the sinusoidal signal and the entire original EEG, the correlation coefficient is computed between the original EEG in a defined band and the remaining filtered target frequencies. There is no need to construct a series of sinusoidal signals and detection accuracy is irrelevant to the initial phase of SSVEP.

Any method of SSVEP extraction is sensitive to the SSVEP power itself. The same rule applies for the PS, SOB and CC methods. This is why a positive correlation can be observed between the signal to noise ratio (SNR) and the detection accuracy. The most important task in a BCI therefore, is to improve the power of SSVEP, except when finding a valid extraction method.

The absolute power of SSVEP is also dependent on other extraneous factors such as attention or viewing angle, except for stimulus intensity. Therefore, the absolute power of SSVEP corresponding to a certain frequency may vary within a range even if the same stimulus is used. In some situations, the absolute power of SSVEP may not be strong enough. Thus, if the power of background EEG increases to a certain extent, the relative power may be below the threshold, which leads to false detection. Similarly, if the power of background EEG decreases to a certain extent, a certain frequency with fluctuating power of a high value can be detected as SSVEP. In other words, relative power is sensitive to the power of background EEG. In the PS method, relative power is often used as an indicator of SSVEP. Therefore, the PS method is sensitive to the background EEG. In the SOB method, in order to decrease sensitivity to background EEG, the band selection of background EEG is very important when constructing a new ‘S1’ signal. SSVEP power is very small compared to the total power of background EEG. In consequence, if the whole band of EEG is chosen in the SOB method, the correlation coefficient holds almost unvaried, even if the strong target frequency is filtered out. This leads to a false detection. To prevent this, some strong background EEG is filtered beforehand. The remaining band of background EEG needs to be broad enough to prevent the average power of the remaining band from fluctuating too much when some components vary over a wide range. If not, some segments with a larger fluctuation of a certain frequency that is not SSVEP, might be detected as the SSVEP of this frequency, decreasing detection accuracy.

The filter parameters given in this work are empirical. In a real BCI, they can be adjusted to an optimal value. For example, to avoid a strong α signal, the signal from 8–12 Hz should filter out. 8.33 Hz and 12.5 Hz stimuli were used in this work, according to the SOB method, the near band background signal should be maintained so that only the background signal from 9 Hz to 11 Hz is filtered out. The filter in this work is based on the frequency domain. Although FFT and IFFT can take up more time, this consumption (10 ms or so) is much shorter compared to the signal length and can therefore be applied in a simple BCI system with a few stimuli. In fact, other kinds of filters in the time domain such as FIR or IIR can also be selected. We have tested this kind of filter and similar detection accuracy can be achieved. In a complicated BCI system including dozens of target frequencies, time consumption for filtering is too long and as such filters in time domains such as FIR or IIR are preferred.

First type accuracy is influenced mainly by the power of SSVEP itself. This is the same for the PS, SOB and CC methods. According to the standards of SSVEP threshold selection, SSVEP power is normally much higher than the threshold. However, for some particular reasons (e.g. if the subject has not paid enough attention to the stimulus or stared at the stimulus at an incorrect angle), SSVEP power will decrease dramatically, reducing first type detection accuracy. In this study, there was a big difference in first type accuracy for the same person during different periods. This is because this experiment was not a real BCI and the subjects would sometimes divert their attention or move the viewing angle, decreasing SSVEP power. In fact, when applying FFT for a long enough period, such as 4–5 s length, a large fluctuation in SSVEP power was observed for different segments. The subjects diverting their attention or moving the viewing angle led to a clear inter-subject difference of SSVEP intensity. If treating all subjects as one group, first type detection accuracy varies acutely between subjects, which is not a normal distribution, and the inter-subject differences conceal the differences between methods. As such, there is no significant difference in first type accuracy between the methods. Dividing the subjects into groups according to their SSVEP intensity, results in a significant difference between the methods, for the groups with middle or low strength SSVEP. This suggests that the SOB method is more valid than the PS and CC methods in a situation where SSVEP is weak.

Second type accuracy is influenced mainly by background EEG. In an SSVEP-based BCI, normally many frequencies are adopted simultaneously. For a segment including a certain frequency SSVEP that is strong enough, SSVEP can usually be detected. However, for other frequencies, due to power fluctuations, they may sometimes exceed the threshold and be detected as SSVEP. This would lead to false second type detection. Since the SOB method has lower sensitivity to background EEG compared to the PS method, second type accuracy of the SOB method is significantly higher than that of the PS method. Usually, random noise is asynchronous and thus the CC method is sensitive to the initial phase. Therefore, asynchronous noise can rarely result in a large correlation coefficient, and the second type accuracy of the CC method is similar to that of the SOB method.

First type accuracy and second type accuracy are both important for a real BCI. A valid extraction method should have high accuracies for both. If not, a balance should be found between the two type accuracies. This balance is closely related to the threshold. If the threshold is too low, first type accuracy will be very high. However, excessive noise will be incorrectly detected as SSVEP, subsequently decreasing second type accuracy. In contrast, if the threshold is too high, background noise will not exceed the threshold and high second type accuracy can be obtained. However, some weak SSVEPs cannot be detected under this high threshold, which can decrease first type accuracy. In this study, in order to compare the validity of the three methods qualitatively, the threshold was built based on a spontaneous EEG. This is reasonable because in a BCI, some target frequency components vary according to the subject’s attention, while other background components hold at a similar level to the spontaneous EEG. The threshold based on a spontaneous EEG can adapt to this situation for second type detection. The balance parameter, for example, the 90% first type accuracy for spontaneous EEG in this study, can be adjusted to the designer’s preference.

The objective of this work is to develop a valid SSVEP extraction method for a short-time period, so we mainly focused on the detection for 1s-length segment. On the other hand, in order to understand the performance of the different methods changing with the signal duration, we also detected SSVEP in 3s-length segment with different methods. The results show that the detection accuracy of PS increases with the rise of duration, while for the other two methods, the detection accuracy has no significant improvement with the increase of duration. In the SOB and CC methods, the SSVEP detection is based on the computing of correlation coefficients, while this coefficient is almost irrelative to the signal length, so the detection accuracy would not vary with the increase of duration. The SSVEP is synchronous, but the background noise is asynchronous. When using FFT to get the power spectrum of the evoked EEG, the synchronous signal would be enhanced as the duration increases, while the asynchronous signal would almost keep the same level as the duration increases. So the detection accuracy of a longer segment is higher than that of shorter segment in the PS method. Although the PS method has a high detection accuracy for the longer segment, it is not suitable for a real-time system for its long time consumption.

## Conclusion

The SOB method is insensitive to the initial phase of SSVEP. Compared to the PS method, the SOB method has a lower sensitivity to background EEG. Although the SOB method and the CC method both make use of the correlation coefficient, the mechanism is completely different. When applying the SOB method for detecting the SSVEP for a short period such as 1s-length, higher detection accuracy than that produced by the PS and CC method can be acquired. All these features suggest that the SOB method can be easily applied in a real-time BCI.
